# Suppression of the DNA repair enzyme NEIL2 promotes persistent inflammation and genomic damage in subjects with stable COPD and during severe exacerbations

**DOI:** 10.21203/rs.3.rs-4849668/v1

**Published:** 2024-09-03

**Authors:** Victor J Cardenas, Justin B Seashore, Nisha Tapryal, Moe Ameri, Rosalinda Rivera, Kabir Sharma, Tapas Hazra

**Affiliations:** University of Texas Medical Branch; Kaiser Permanente Northern California, Vacaville Medical Center; University of Texas Medical Branch; University of Texas Medical Branch; University of Texas Medical Branch; University of Texas Medical Branch; University of Texas Medical Branch

**Keywords:** DNA repair, COPD, cancer, genomic damage, airway inflammation, eosinophils, NEIL2

## Abstract

**Background.:**

Chronic obstructive pulmonary disease (COPD) is a chronic inflammatory airway disease that is an independent risk factor for lung cancer. NEIL2, a DNA glycolase involved in DNA repair during transcription, has also been associated with an increased incidence of malignancies in humans. NEIL2 knockout mouse models have demonstrated increased inflammation and oxidative DNA damage in the lungs after exposure to an inflammatory insult, but data are lacking regarding NEIL2 function in individuals with stable COPD and during severe acute exacerbations of COPD (AECOPD). We investigated whether NEIL2 levels and oxidative DNA damage to the transcribed genome are altered in individuals with stable COPD and AECOPD.

**Methods:**

The study was conducted at a single center in the US. Eligible subjects underwent a one-time 30 cc venous blood draw. The population consisted of 50 adults: 16 with stable COPD, 11 hospitalized for AECOPD, and 23 volunteers. We analyzed blood leukocytes for NEIL2 mRNA and DNA damage by RT–qPCR and LA–qPCR, respectively, in all groups. Plasma levels of seven biomarkers, CXCL1, CXCL8, CXCL9, CXCL10, CCL2, CCL11 and IL-6, were analyzed in the COPD groups using a magnetic bead panel (Millipore^®^).

**Results.:**

The NEIL2 mRNA levels were lower in individuals with stable COPD and AECOPD than in controls (0.72 for COPD, p = 0.0289; 0.407 for AECOPD, p = 0.0002). The difference in NEIL2 mRNA expression between the stable COPD group and AECOPD group was also statistically significant (p < 0.001). The fold change in DNA lesions per 10 kb of DNA was greater in the stable COPD (9.38, p < 0.0008) and AECOPD (15.81, p < 0.0004) groups than in the control group. The difference in fold change was also greater in the AECOPD group versus stable COPD p < 0.0236). Biomarker levels were not significantly different between the COPD groups. NEIL2 levels were correlated with plasma eosinophil levels in the stable COPD group (r = 0.737, p < 0.0027).

**Conclusions.:**

NEIL2 mRNA levels are significantly reduced in COPD subjects and are associated with increased DNA damage and inflammation. These results reveal a mechanism that promotes persistent airway inflammation and oxidative genomic damage and increases the risk of malignancy in this population.

## BACKGROUND

Chronic obstructive pulmonary disease (COPD) is a major health burden in the United States and is the third leading cause of death at 148,000/per year, with an estimated cost of $50 billion per year (1). COPD is characterized by chronic airway inflammation, fixed airway obstruction, and variable rates of progression. Some patients experience episodic worsening of symptoms (cough, increased sputum production, breathlessness, etc.) described as acute exacerbation of COPD (AECOPD). Frequent and more severe exacerbations often result in hospitalizations and are associated with more rapid progression of the disease (2). COPD has been identified as an independent risk factor for the development of lung cancer, with an estimated odds ratio of 1.6–2 (3, 4). Additionally, the presence of acute exacerbations has also been associated with an increased risk of lung malignancy (5). Lung cancer is the leading cause of death due to malignancy in the United States, with an estimated 127,000 deaths per year, greater than all deaths due to breast, prostate and colon cancer combined (3). However, the link between COPD and lung cancer has not been fully delineated. There are a plethora of reports showing that chronic airway inflammation in COPD subjects is also associated with systemic inflammation characterized by leakage of reactive oxygen species (ROS) and cytokines directly into the peripheral blood that further preactivate blood leukocytes (6–9). Indeed, substantially increased systemic inflammatory markers, such as leukocytes, interleukin (IL)-6, IL-8, C-reactive protein, fibrinogen and tumor necrosis factor alpha (TNF-α), have been reported in COPD subjects compared to healthy individuals (9–11). Additionally, NO and O2^−^ released from neutrophils or macrophages during inflammation not only increase the number of mutagenic DNA lesions, such as 8-nitroguanine and 8-oxodG but also impair the DNA repair machinery by inhibiting a number of important DNA repair enzymes (12–14). In cells, oxidative DNA damage is repaired by base excision repair (BER). The BER pathway involves a multiprotein complex that utilizes one of five DNA glycosylases that recognize single oxidized bases and initiates the process of removal and replacement with the appropriate nucleotide. NEIL2 is a unique DNA glycosylase that removes single oxidized bases during transcription when DNA is in a bubble structure (15). A reduction in NEIL2 activity has been associated with increased oxidative damage in the transcriptionally active genome in animal models (16). Reduced or aberrant activity of NEIL2 has also been linked to a variety of human malignancies, including lung cancer (17, 18). Recently, we showed a protective anti-inflammatory role of NEIL2 in TNF-α-induced lung inflammation (19). Additionally, pathogenic bacterial infections, such as *H. pylori* and Fusobacterium, and viral infections such as respiratory syncytial virus (RSV) and severe acute respiratory syndrome coronavirus 2 (SARS-CoV-2), significantly downregulate NEIL2, with a consequent increase in the expression of inflammatory cytokines (20–23). Furthermore, bacterial or viral infections are the most frequent causes of exacerbations in COPD patients. However, the link between COPD and oxidative DNA damage and the role of NEIL2 therein have never been explored.

We propose that NEIL2 activity provides a link between persistent airway inflammation and increased DNA damage, which are risk factors for lung malignancy and several comorbidities in COPD patients. Thus, we hypothesized that low levels of NEIL2 result in persistent inflammation and increased oxidative damage to the transcriptionally active genome in human subjects with COPD. To test our hypothesis, we measured NEIL2 mRNA levels and oxidative damage in circulating blood leukocytes in subjects with stable COPD and severe AECOPD.

## METHODS

Our study is a single-center cross-sectional cohort study. The subjects were divided into three groups. Group 1 consisted of patients seen in the UTMB Pulmonary Outpatient clinics for routine follow-up visits. The inclusion criteria for subjects were evidence of chronic airflow obstruction on spirometry (FEV1/FVC < 80% predicted), stable symptoms for more than 3 months without exacerbations, and no recent use of oral steroids for any reason. Group 2 consisted of COPD patients who were hospitalized at UTMB hospitals for a primary diagnosis of AECOPD (increased breathlessness, cough and/or sputum production). Subjects were excluded if there was evidence of pneumonia, sepsis, or decompensated heart failure. Group 3 consisted of normal volunteers without evidence of intrinsic lung disease or acute inflammatory process/infection. Demographics and baseline data were obtained from the electronic medical record.

### Procedures

The subjects underwent venipuncture with one-time removal of 30 ccs of whole blood. Blood was separated into plasma and buffy coats at the UTMB Clinical Research Center, frozen at −80°C within four hours of sampling and maintained at that temperature until the assays were performed.

### Real-time quantitative polymerase chain reaction (RT–qPCR)

Total RNA was isolated from blood samples via the PAXgene Blood RNA Kit (Qiagen) according to the manufacturer’s protocol. Up to 2 μg of RNA was used to synthesize cDNA using a PrimeScript^™^ RT Kit with gDNA Eraser (TaKaRa), and qPCR was carried out using TB Green^™^ Premix Ex Taq^™^ II (Tli RNase H Plus; TaKaRa) in an Applied Biosystems^™^ 7500 Real-Time PCR System with thermal cycling conditions of 94°C for 5 min (94°C for 10 s and 60°C for 1 min) for 40 cycles and 60°C for 5 min. The target mRNA levels were normalized to that of glyceraldehyde 3-phosphate dehydrogenase (Gapdh). In each case, qPCR was performed with DNase-treated RNA samples without reverse transcriptase to rule out genomic DNA contamination.

### DNA damage: Long amplicon quantitative PCR (LA-qPCR)

Approximately 2 ml of blood was subjected to centrifugation at 2000 × g for 10 min at room temperature, and the buffy coat was collected in a fresh Eppendorf tube. Genomic DNA was extracted using the DNeasy Blood and Tissue Kit (Qiagen) according to the manufacturer’s protocol. The DNA was quantified by Pico Green (Molecular Probes) in a black-bottomed 96-well plate, and 20–30 ng of DNA was used for LA-qPCR assays as described earlier (Chakraborty et al. 2020) using LongAmp Taq DNA Polymerase (NEB). The transcribed gene HPRT1 (~10 kb) was amplified using the appropriate oligos. The final PCR conditions were optimized at 94°C for 30 s; 24 cycles of 94°C for 30 s, 58°C for 30 s, and 65°C for 10 min; and 65°C for 10 min. Since the amplification of a small region is independent of DNA damage, a small DNA fragment (~200–400 bp) from HPRT1 was also amplified for normalization of the long amplicon. The amplified products were then visualized on gels using ethidium bromide, and the intensity of the bands was quantified with ImageJ software (NIH). The relative extent of DNA damage was determined by calculating the intensity of bands, and lesion frequencies were derived from the expression −ln(A_T_/A_C_), where A_T_ is the amplification of the test sample (stable COPD or AECOPD), and A_C_ is the amplification of a nondamaged control (24).

### Cytokine Testing

Plasma samples were tested using the MILLIPLEX^®^ Human Cytokine/Chemokine/Growth Factor Magnetic Bead Panel A (Millipore^®^). A seven-point serial dilution standard curve was constructed by reconstituting the lyophilized standards provided in the kit. The plasma samples were tested undiluted and treated as instructed in the kit. A 96-well plate was loaded with the standard curve and positive controls, and samples were then tested for CXCL1, CXCL8, CXCL9, CXCL10, CCL2, CCL11, and Il-6. Bead fluorescence readings were captured using the Bio-Plex 200 System (Bio-Rad^®^).

### Statistics

Demographic variables were analyzed using Fisher’s exact test for categorical variables and unpaired t tests for continuous variables. Two-sided unpaired Student’s t test (http://www.ruf.rice.edu/~bioslabs/tools/stats/ttest.html) and online MedCalc statistical software (https://www.medcalc.org/calc/comparison_of_means.php) were used for analysis of the statistical significance between two sets of data for mRNA expression and DNA oxidative damage values. Cytokine levels were assessed using the Mann–Whitney Rank Sum test for nonnormally distributed data. Pearson’s correlation was used for analysis of NEIL2 mRNA levels and cytokine and eosinophil levels. Statistical significance was set at p< 0.05. Statistics were generated using SigmaPlot 15 software (SYSTAT Software, Inc., San Jose, CA).

## RESULTS

Sixteen subjects were included in the stable COPD group, 11 subjects were included in the severe COPD exacerbation group, and 23 were included in the control group. Demographics and baseline data are recorded in Table 1 below. The baseline FEV1 was significantly lower in the AECOPD arm.

### DNA glycosylase expression and DNA damage

**Figure 1:**
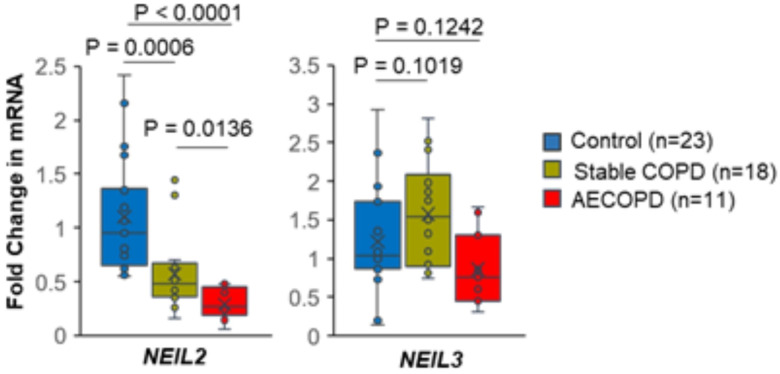
DNA glycolase levels by group

We tested the mRNA expression of the DNA glycosylases NEIL2 and NEIL3, which are DNA base-specific enzymes that initiate the first step of the BER pathway, in blood cells from COPD subjects (n = 29), including 11 subjects suffering from AECOPD, compared to those from healthy individuals (n = 23). We observed an approximately 48% decrease (P = 0.0006) in the NEIL2 mRNA level in the stable COPD group compared to the control cohort (Fig. 1). Furthermore, NEIL2 expression was significantly lower in the AECOPD cohort than in both the control (over 73%; p < 0.0001) and stable COPD (49%; p = 0.0136) cohorts. Conversely, no significant change in NEIL3 was observed among the groups. Overall, the expression of the DNA repair enzyme NEIL2 is significantly decreased in stable COPD subjects and is further decreased in subjects suffering from AECOPD.

**Figure 2:**
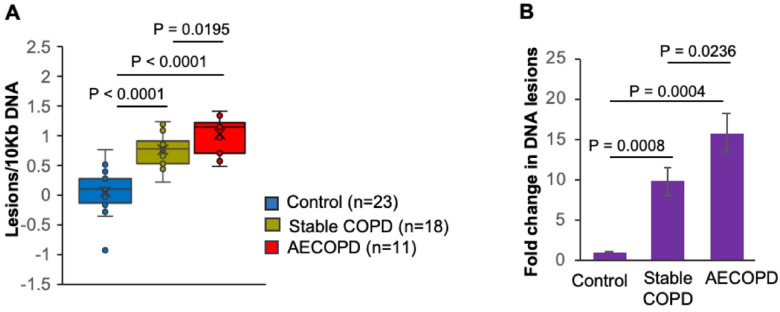
DNA damage by groups

NEIL2 has been shown to protect the transcribing genome by participating in transcription-coupled base excision repair (TC-BER) (15, 16); nonetheless, a decrease in NEIL2 leads to the accumulation of DNA lesions, especially in the transcribing genome. Thus, in light of the diminished NEIL2 levels in COPD subjects, we examined DNA damage in blood cells using the model transcribed gene HPRT1, and indeed, we observed a significant increase in DNA damage in stable COPD and AECOPD subjects compared to that in the control group (Fig. 2A). Compared with those of controls, stable COPD subjects showed a 9.78-fold (P = 0.0008) greater accumulation of DNA lesions, whereas the frequency of DNA lesion accumulation was significantly greater (15.81-fold, P = 0.0004) in AECOPD subjects (Fig. 2B). Notably, compared with stable COPD subjects, AECOPD subjects exhibited ~ 1.6-fold (P = 0.0236) greater oxidative DNA damage, which is in accordance with the significantly lower levels of NEIL2 in AECOPD subjects than in stable COPD subjects or healthy controls (Fig. 1).

### Cytokine levels and systemic inflammation

**Figure 3:**
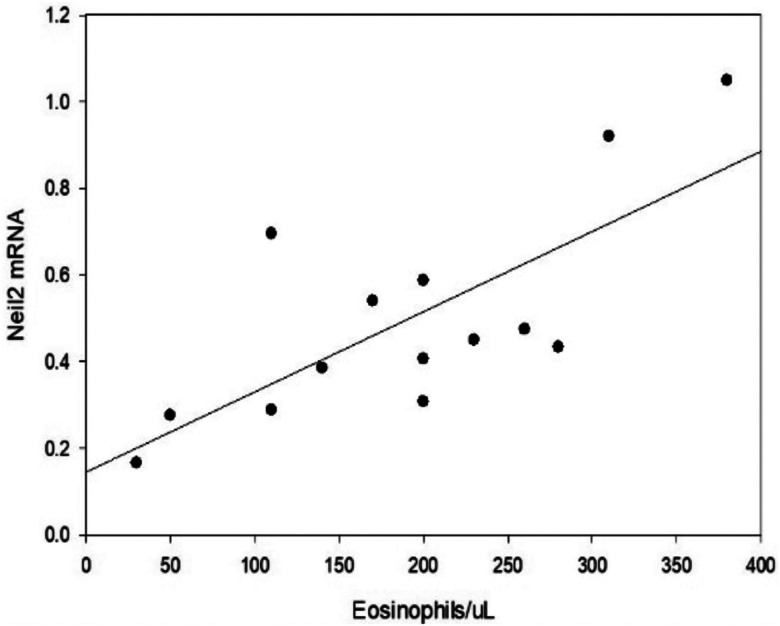
Correlation between Neil2 mRNA levels and blood eosinophils in stable COPD subjects

There was no correlation between cytokine levels and NEIL2 mRNA levels across groups, Table 2. Furthermore, there was no significant difference in cytokine levels between the COPD groups, Table 3. There was a significant positive correlation between NEIL2 and blood eosinophil levels (r = 0.737, p = 0.00265) in stable COPD subjects (Fig. 3). AECOPD subjects who had received parenteral corticosteroids at the time of sampling were excluded.

## DISCUSSION

Inadequate DNA repair is implicated in the pathogenesis of chronic obstructive pulmonary disease (25); however, the mechanisms that underlie inadequate DNA repair in COPD are poorly understood. In the present study, we investigated the expression levels of key enzymes of the base excision repair pathway that initiate the repair of oxidatively damaged bases in cohorts of stable COPD subjects and subjects suffering from AECOPD. The levels of NEIL2 mRNA were significantly decreased in stable COPD subjects and further reduced in the AECOPD group. Reduced levels of NEIL2 were also associated with significantly increased DNA damage in the transcriptionally active genome in both stable COPD and AECOPD subjects compared to that in controls, with significantly increased damage in the AECOPD cohort compared to that in stable COPD group. This finding is consistent with findings in NEIL2 knockout mice that demonstrated cumulative oxidative damage in the transcriptionally active genome over time (16).

Reduced NEIL2 levels in stable subjects without evidence of exacerbation are associated with persistent oxidative damage that could result in genome instability and dysfunctional transcription and contribute to the development of malignancy. A reduction in NEIL2 activity caused by dysfunctional polymorphisms has been associated with an increased risk of malignancy (17, 18). Acute exacerbations result in further reductions in Neil 2 levels, suggesting that this may be a mechanism that contributes to the increased risk of malignancy in COPD subjects with frequent exacerbations.

The most common cause of exacerbation is infection, which may explain the further suppression of Neil 2 levels and increased inflammation, as observed in RSV- and SARS-CoV-2-infected animal models (22, 23). Alternately, this group may have lower chronic depression of NEIL2 levels, which in turn increases their susceptibility to acute exacerbations and increased genomic damage. Although there was no significant correlation between the degree of airflow obstruction and NEIL2 mRNA, those subjects with AECOPD had a significantly lower FEV1 than did those in the stable group. Additionally, as reported previously, NEIL2 plays an essential role in maintaining immune response homeostasis, where NEIL2 blocks unwarranted NF-kB-mediated proinflammatory gene expression and protects hosts from an uncontrolled inflammatory response (19, 23). Dysregulation of inflammation due to the production of abnormal proteins could also impair the healing process and lead to more rapid disease progression. The cause of the reduction in NEIL2 in stable COPD subjects is unclear. There was no clinical evidence of ongoing infection or a history of lung malignancy in this group. However, the adverse effect on oxidative damage in the transcriptional genome was quite clear. As this was a cross-sectional study, we are unable to determine whether the levels might recover with time, but given that most were former smokers and had no recent exacerbations, this seems unlikely.

A recent meta-analysis using two large COPD cohorts did not demonstrate the predictive value of any cytokine for acute exacerbations (26). Similarly, we examined 7 cytokines, including IL6, CXCL 1, 8, 9, 10, CCL2, and CCL11, in our COPD population and found no significant differences between the COPD groups. The positive relationship between blood eosinophil levels and NEIL2 expression was unexpected. A recent analysis of blood eosinophil levels in the Spiromics COPD cohort revealed that GOLD D subjects with eosinophil counts less than 100/pl who did not receive steroids had more exacerbations and a more rapid decline in lung function (27). Therefore, blood eosinophils may be an indicator of low NEIL2 expression, which results in persistent inflammation and worsening genomic damage. The relationship between NEIL2 and eosinophil levels needs further investigation.

Our study is limited by its cross-sectional nature and use of circulating blood leukocytes. A follow-up study examining sampling of airway epithelium and leukocyte subgroups may clarify the role of the NEIL2-BER pathway. Given the low levels we detected, the use of recombinant NEIL2 as a therapeutic intervention is possible. We previously showed that recombinant NEIL2 modulates the inflammatory response by inhibiting NF-kB-mediated gene expression and decreasing viral replication (19, 22, 23). Thus, restoring NEIL2 levels could improve therapy for severe exacerbations, slow the progression of COPD in subjects at risk for a more rapid decline in lung function and/or frequent exacerbations and reduce the risk of malignancy in certain subgroups.

## CONCLUSIONS

Low levels of NEIL2 mRNA identify a mechanistic link between persistent airway inflammation and oxidative genomic damage in COPD patients. Potential consequences include an increased risk of malignancy, severe exacerbations, and an increased rate of lung function deterioration.

## Figures and Tables

**Table 1:** Baseline characteristics of individuals with Chronic Obstructive Pulmonary Disease (COPD) and Acute Exacerbation of COPD (AECOPD)

Variable	COPD (n = 16) n (%) or mean (±SD)	AECOPD (n = 11) n (%) or mean (±SD)	p-value
**Age (years)**	62.6 (3.5)	66.5 (4.9)	0.376
**Sex (n)**			
Male	4 ()	3 ()	0.900
Female	12 ()	8 ()	
**Race (n)**			
White	16 (100.0)	9 ()	0.080
Black	0 (0.0)	2 ()	
**Current Smokers (n)**	4 (25.0)	3 (27.0)	0.900
**Total pack years (mean)**	42.929 (9.3)	46.8 (26.4)	0.751
**Forced Vital Capacity (FVC) (L)**	1.96 (0.8)	2.29 (0.8)	0.355
**Forced Expiratory Volume in one second (FEV**_**1**_ **) (L)**	1.46 (0.7)	0.88 (0.4)	0.029
**FEV**_**1**_ **/FVC (%)**	51.5 (12.7)	40.0 (17.92)	0.094
**Diffusing Capacity (DLCO) (ml/min/Hg)**	15.87 (5.6)	9.66 (4.8)	0.050
**Eosinophils (cells/μl)**	190.7 (98.6)	X	n/a

Student’s t-test, significance p < 0.05

**Table 2:** Pearson Product Correlation between plasma cytokine levels and NEIL2 mRNA levels

	CXCL1	CXCL8	CXCL9	CXCL10	CCL2	CCL11	IL 6
NEIL2	0.256	.0678	−0.307	−0.366	−0.287	0.051	−0.0571
P value	0.263	0.742	0.127	0.0661	0.155	0.804	0.791

Significance p < 0.05

**Table 3:** Plasma cytokine levels in blood leukocytes (pg/ml) by group

	Median Value	25th %	75th %	P value
**CXCL1**				
**COPD**	5.63	3.650	9.210	
**AECOPD**	6.93	2.560	11.480	p = 0.481
**CXCL8**				
**COPD**	1.95	0.857	3.152	
**AECOPD**	1.38	0.940	8.490	p = 0.767
**CXCL9**				
**COPD**	2061.945	1136.225	3155.440	
**AECOPD**	2802.91	1142.130	4386.410	p = 0.521
**CXCL10**				
**COPD**	175.25	109.020	309.325	
**AECOPD**	255.02	119.810	399.890	p = 0.167
**CCL2**				
**COPD**	280.595	183.063	302.445	
**AECOPD**	171.3	115.040	371.950	p = 0.657
**CCL11**				
**COPD**	64.75	47.990	82.415	
**AECOPD**	62.84	35.080	118.190	p = 0.474
**IL 6**				
**COPD**	4.015	1.735	5.525	
**AECOPD**	4.88	2.448	11.470	p = 0.257

Mann-Whitney Rank Sum test, significance set at p< 0.05

## Abbreviations

COPD: chronic obstructive pulmonary disease
AECOPD: acute exacerbation of COPD
GOLD: Global Initiative for Chronic Obstructive Lung Disease
NEIL2: Nei-like 2
RT–qPCR: Real-time quantitative polymerase chain reaction
LA-qPCR: Long amplicon quantitative PCR
CXCL: C-X-C motif chemokine ligand
CCL: C-C motif chemokine ligand
IL: interleukin
DNA: deoxynucleic acid
mRNA: messenger ribonucleic acid
TC-BER: transcription coupled-base excision repair.
RSV: Respiratory Syncytial Virus
SARS-CoV-2: severe acute respiratory syndrome coronavirus 2
NF-kB: nuclear factor kappa B
TNF-α: tumor necrosis factor alpha
Gapdh: glyceraldehyde 3-phosphate dehydrogenase
FEV1: Forced expiratory volume in 1 second.
FVC: Forced vital capacity
